# A learnable parallel processing architecture towards unity of memory and computing

**DOI:** 10.1038/srep13330

**Published:** 2015-08-14

**Authors:** H. Li, B. Gao, Z. Chen, Y. Zhao, P. Huang, H. Ye, L. Liu, X. Liu, J. Kang

**Affiliations:** 1 Institute of Microelectronics, Peking University, Beijing 100871, China

## Abstract

Developing energy-efficient parallel information processing systems beyond von Neumann architecture is a long-standing goal of modern information technologies. The widely used von Neumann computer architecture separates memory and computing units, which leads to energy-hungry data movement when computers work. In order to meet the need of efficient information processing for the data-driven applications such as big data and Internet of Things, an energy-efficient processing architecture beyond von Neumann is critical for the information society. Here we show a non-von Neumann architecture built of resistive switching (RS) devices named “iMemComp”, where memory and logic are unified with single-type devices. Leveraging nonvolatile nature and structural parallelism of crossbar RS arrays, we have equipped “iMemComp” with capabilities of computing in parallel and learning user-defined logic functions for large-scale information processing tasks. Such architecture eliminates the energy-hungry data movement in von Neumann computers. Compared with contemporary silicon technology, adder circuits based on “iMemComp” can improve the speed by 76.8% and the power dissipation by 60.3%, together with a 700 times aggressive reduction in the circuit area.

For decades, modern computers have been playing the central role in human society processing massive amounts of information every day. Behind every single operation our computers execute is the well-known von Neumann computer architecture^1^. In this architecture, the computing and memory units are separated and connected via buses, through which the instruction codes and computing data are consecutively transported between processors and memories. However, every joule of energy used for moving data between memories and processors consumes the limited budget, leaving less energy available for actual computation in our computers. This kind of frequent energy-hungry movement is thereby regarded as the “von Neumann bottleneck”^2^. Today, the need for energy-efficient information systems is as great as ever, covering various domains from big data processing and Internet of Things to wearable healthcare devices. Therefore, making a fundamental change from the bottom of the current computer architecture is essential to support diverse societal applications^3^. Alternative approaches such as logic-in-memory^4^,^5^,^6^ might mitigate von Neumann bottleneck due to the colocation of logic and memory units, but the inherent boundary between these two parts in the whole system could still limit the potential of energy saving^7^. Aiming at breaking the bottleneck in both device and architecture levels, we develop and demonstrate a non-von Neumann architecture named “iMemComp” based on resistive switching (RS) devices.

## Results

### “iMemComp” architecture

**Figure 1:**
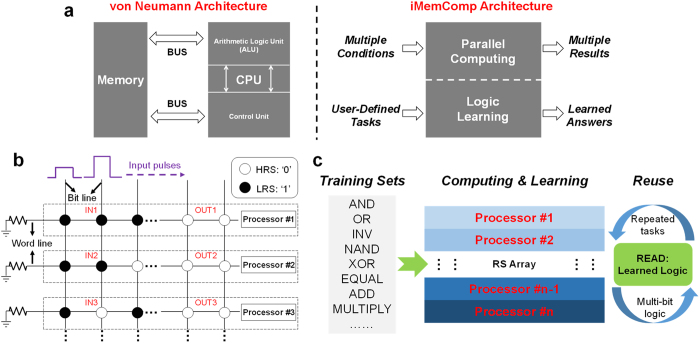
“iMemComp” architecture. (**a**) Compared with von Neumann architecture where central processing unit (CPU) and memory are separated by buses, “iMemComp” unifies both logic and memory functions realized by resistive switching (RS) devices, offering new features such as parallel computing and logic learning. (**b**) The “iMemComp” is entirely built upon crossbar RS arrays. Parallel computing in “iMemComp” capitalizes on the structural parallelism of crossbar arrays. Different input combinations stored at multiple rows are simultaneously involved in the computation under pulse-train operations, and therefore, various results are obtained and stored *in situ*. In this context, a single row represents an independent processor. All of the processors together serve as *in situ* memories meanwhile. (**c**) “iMemComp” is equipped with “logic learning” capability owing to the nonvolatile nature of RS devices. Boolean logic and other user-defined functions train the RS arrays at the time of computation, leaving the answers memorized by RS cells. These learned logic functions, which are easy to readout from crossbar arrays through decoding, can be reused for multi-bit logic and large-scale repeated tasks.

Compared with the von Neumann architecture with separated modules, “iMemComp” uses a unified core to perform both memory (“Mem”) and computing (“Comp”) tasks, with the capability of parallel computing, learning and memorizing (“intelligent”) user-defined logic functions *in situ* (Fig. 1a). Instead of using modern complementary metal-oxide-semiconductor (CMOS) transistors, “iMemComp” is built upon RS devices, a kind of sandwich-like emerging device whose resistance can be modulated via applying external voltage (Supplementary Fig. S1)^8^,^9^,^10^,^11^. Owing to a series of advantages such as simple structure, low operation power, high switching speed and great scalability^12^,^13^,^14^,^15^,^16^, RS devices have been widely studied for both future memory technology^12^,^13^,^14^,^15^,^16^,^17^,^18^,^19^,^20^ and computing applications^12^,^21^,^22^ where the state variable is cell resistance rather than voltage or charge in traditional logic circuits^23^. By combining the nonvolatile memory and Boolean logic functions, “iMemComp” enables new features beyond von Neumann architecture: (i) parallel computing and (ii) logic learning (Fig. 1b,c). For (i) parallel computing, specifically, we employ the crossbar RS arrays as the building blocks in “iMemComp” (Fig. 1b). During the operation of RS arrays, data are stored within RS cells in a nonvolatile form as device resistance, and logic functions are performed via pulse-train operations at bit lines (Supplementary Fig. S2). Inspired by the structural parallelism of crossbar arrays, we have found that this kind of high-density circuit structure can lead to parallel computing ability. Prior to computing operations, different input combinations (IN) are stored independently at multiple rows along word lines in the RS array. With pulses applied at bit lines to execute computing, computation can be carried out in parallel among different rows to yield multiple results (OUT). Hence, in a single array we have multiple processors, and all of them serve as memories as well (Fig. 1b); for (ii) logic learning, once user-defined logic functions are computed in RS arrays, the results together with input combinations will be stored *in situ* and remain nonvolatile. Regarding basic Boolean logic and other user-defined functions as training sets, RS arrays are able to learn the logic operations. Moreover, the answers that have been remembered by RS arrays can be read out for the following multi-bit computation and large-scale repeated tasks (Fig. 1c).

### Functional completeness and experimental features

**Figure 2:**
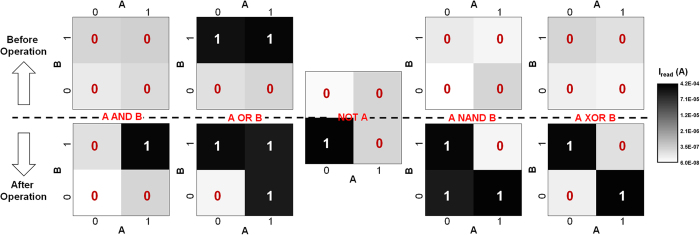
Experimental demonstration of functional completeness in “iMemComp”. AND, OR, INVERT (AOI) together with NAND and XOR logic functions are demonstrated (from left to right). The states of the OUT cells before and after operations are read out as current values (high current represents ‘1’ and low current represents ‘0’) shown by the gray-scale maps. Despite the variations around ‘0’ due to the variability of HRS, the distinct computing results verify the feasibility of nonvolatile logic operations in “iMemComp”. The functionally complete AOI logic, NAND, and XOR can serve as building blocks for more complex computing tasks.

**Figure 3:**
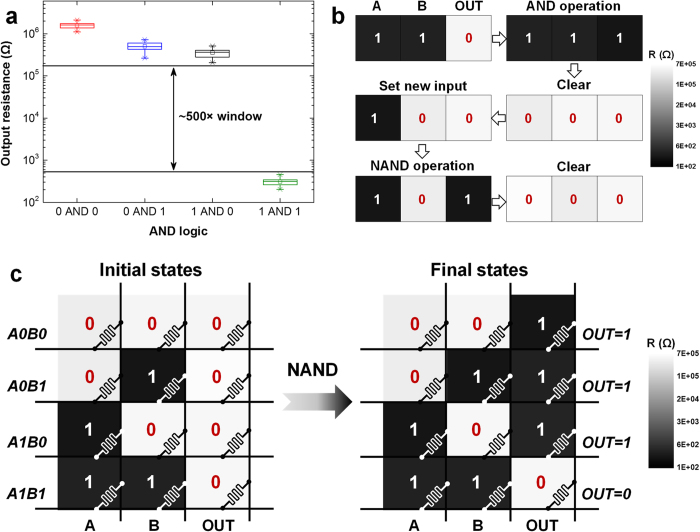
Experimental features: reproducibility, reconfigurability, and parallelism. (**a**) Box plot of the measured AND operations as a proof of reproducibility. Each AND operation with a certain input combination is repeated for 20 cycles. The computation in ‘iMemComp’ relies more on the resistance window between LRS and HRS than the specific resistance values of input or output. Therefore, the computation is well reproducible despite the variability of HRS and LRS. (**b**) Measured resistance evolution of input and output RS cells from AND operation to NAND operation. The logic functions carried out in “iMemComp” are reconfigurable by clearing previous states (RESET) and performing new pulse-train operations, without changing circuit topology like CMOS circuits. (**c**) Measured parallel NAND logic in a crossbar RS array. With single pulse-train operation for NAND logic, computation along multiple rows with different input combinations is correctly conducted in parallel, yielding the complete truth table of NAND logic.

We first demonstrate a suite of logic functions including AND, OR, INVERT (AOI), NAND and XOR, together with memory operations as a proof of functional completeness. As the elementary functions for other complex computing tasks, AOI, NAND and XOR logic functions can be realized within four RS cells (Supplementary Fig. S3). Figure 2 shows the complete truth-table output results of AOI, NAND and XOR operations. Logic ‘1’ is assigned to low resistance states (LRS) and logic ‘0’ is assigned to high resistance states (HRS). For every kind of logic herein, the states of output cells (OUT) before and after the computation are read out. The correct results shown in Fig. 2 verify both logic and memory functions realized by RS devices. Different from the case of CMOS logic gates where logical output responds to the changing of input during power supply, the nonvolatile logic operations in ‘iMemComp’ are re-triggered by pulse operations at crossbar arrays once input has changed. In a modern central processing unit (CPU) where CMOS transistors serve as digital switches for computing, information flows in a volatile manner since the voltage at nodes cannot be kept without global voltage supply. This fundamental limitation leads to the requirements of registers, memory caches, and external data storage modules. Meanwhile, the data stored in these memories cannot “respond” to computing instructions themselves, which finally causes the energy-hungry communication between CPU and memories. Coming back to Fig. 2, comparing the cell states read out before and after each operation, RS devices serve as a kind of special memories which can directly respond to computing instructions and *in situ* update their nonvolatile states. As shown in Fig. 3, there are three important features of the nonvolatile logic operations in “iMemComp”: reproducibility, reconfigurability, and parallelism. Figure 3a gives the box plot of the measured AND operations, where each operation with a certain input combination is repeated for 20 cycles. Between each cycle, RESET and SET pulses are used to clear the stored data and set up new input. Although there exist resistance variations in HRS and LRS of RS devices, the statistical results reveal that the computation based on RS devices still functions correctly. The HRS/LRS window leads to the distinct logical results after pulse operations (Supplementary Fig. S2). Hence, the measured logical window in Fig. 3a is a result of the resistance window rather than the specific resistance values of individual RS devices. In addition to the repeatability of a certain operation, the logic in ‘iMemComp’ is also reconfigurable, which means the flexible switch between different logic functions is supported. Figure 3b shows the measured resistance evolution of input and output RS cells switching from AND operation to NAND operation. Unlike CMOS logic circuits whose structure and layout vary with the designed functions, here reconfigurable feature of “iMemComp” does not require changing circuit topology. Instead, different logic operations are performed in the same RS array (Fig. 3b), and the switch among logic functions is realized by clearing previously stored data (RESET operation) and applying new operating pulses (Supplementary Fig. S3). In this way, circuit resources can be fully utilized. Moreover, the intrinsic parallelism of “iMemComp” is experimentally demonstrated in Fig. 3c. Initially, all input combinations of A & B are stored in different rows, and output cells store ‘0’. Pulse-train operation for NAND logic (Supplementary Fig. S3) is then carried out at bit lines to trigger the parallel NAND computation. Correct results are *in situ* stored in the OUT cells and read out forming a complete NAND truth table.

### Parallel computing and logic learning

**Figure 4:**
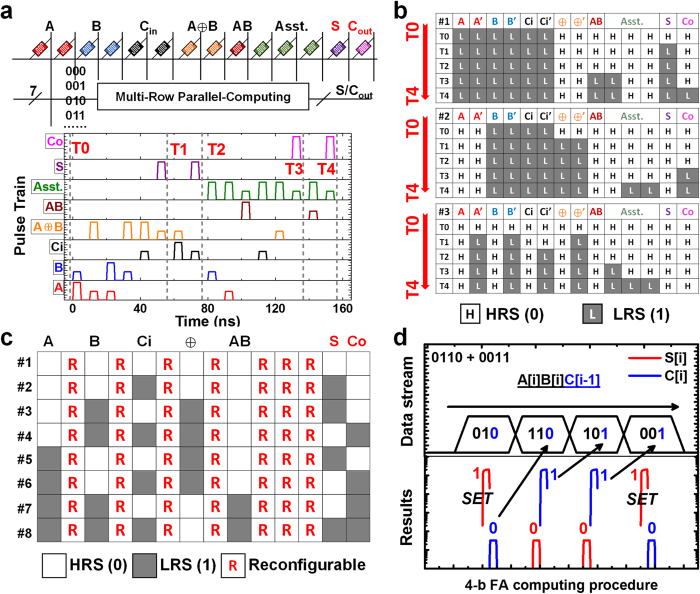
Parallel computing and logic learning. (**a**) Array configuration of full adder (FA) circuits and simulated pulse-train waveforms applied at bit lines for adding operation. Different input combinations (input *A*, *B*, and carry-in *C*_*i*_) are stored at multiple rows in the array, operated under the same pulse-train sequence to perform parallel computing. The output results (sum *S* and carry *C*_*o*_) under various input combinations are stored *in situ* once computation is finished. (**b**) Simulated evolution of the cell states during parallel computing procedure for three typical input combinations (#1: 1 + 1 + 1; #2: 0 + 1 + 1; #3: 0 + 0 + 0). *T*_*0*_ to *T*_*4*_ correspond to the five milestone time nodes marked in the waveform of Fig. 2A, among which *T*_*0*_ and *T*_*4*_ are initial and final stages respectively and *T*_*1*_ to *T*_*3*_are all intermediate stages. (**c**) Complete “knowledge map” learned by FAs after parallel computing. Multiple functions are included in the logic learning, such as AND, XOR and ADD operations. Cells marked by ‘R’ are reconfigurable for other user-defined computing and learning tasks, while the logic functions that are already learned by other cells remain unchanged. (**d**) Simulated computing results of a 4-bit adder capitalizing on logic learning. For each bit of input, binary *A[i]B[i]C[i-1]* is decoded (Supplementary Fig. 7) to find the results stored in the “knowledge map” shown in Fig. 4c. The sum *S[i]* and carry *C[i]* are read out directly for the following bits.

Regarding the functionally complete AOI, NAND and XOR functions together with the features of reconfigurability and parallelism, any other complex logic functions are accessible in “iMemComp” architecture. Adder circuits, for example, play a key role in CPU. A large number of computing tasks are executed relying on the binary adding operations of digital ‘0’ and ‘1’. We then study the design of adder circuits to illustrate the parallel computing and logic learning features (Fig. 4). The adder circuit behaviors are simulated in HSPICE (a commonly used circuit simulator with golden-standard accuracy) using a physics-based SPICE model of RS devices^24^,^25^. The compact model is based on the mechanisms of conductive filament evolution under the co-impact of electrical filed and Joule heating (Supplementary Note & Fig. S4). The model is verified by a series of electrical measurements data (Supplementary Fig. S5), and model parameters including parasitic components are all calibrated by the experimental behaviors of various logic functions demonstrated in Figs 2 and 3 (Supplementary Table S1). Figure 4a shows the multi-row crossbar array configuration of full adder (FA) circuits, where for each FA there are fourteen RS cells along the row. Besides the cells for input and output (input *A, B,* carry-in *C*_*i*_, sum *S* and carry-out *C*_*o*_), there are reduplicative cells for the input and output cells which are designed to guarantee the nonvolatile states of actual input/output cells will not be disturbed during computing. During the pulse-train operation, “A ≈ B” (XOR) and “AB” (AND) are also computed realizing a multi-function circuit, and logic output results are generated in parallel among multiple rows (Supplementary Fig. 6), remaining nonvolatile by the cell resistance (Fig. 4b). Afterwards, the programmed cells along eight rows for all the input combinations form a “knowledge map” storing the information of logic functions of ADD, AND as well as XOR (Fig. 4c). This nonvolatile “knowledge map” can be reused for the following repeated computing tasks based on these three frequent logic functions. Thereby, we have an efficient circle of “logic learning and reusing” in “iMemComp”. Moreover, the reduplicative cells marked by ‘R’ are all reconfigurable (by clearing their states) for other user-defined tasks, and meanwhile the learned logic functions remain reusable for the following tasks. Logic learning is also feasible for multi-bit logic circuits. As a demonstration, a 4-bit adder circuit is designed (Supplementary Fig. 7) and simulated following the logic learning principle (Supplementary Fig. 8). The input combination for each bit is used to decode and select the target row in the “knowledge map” where the matched data are stored after 1-bit adder computation. With the stored results of sum and carry constantly read out for each bit (Fig. 4d), the multi-bit computation can be finished consuming little computational resource.

## Discussion

**Figure 5:**
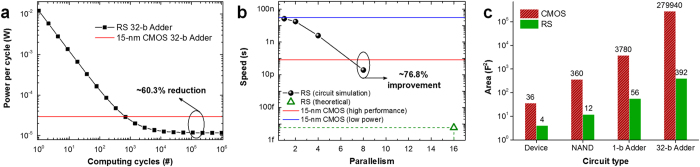
Evaluation of power dissipation, speed, and circuit area. (**a**) Comparison of power dissipation per computing cycle of 32-bit adder circuits based on RS devices and 15-nm CMOS technology. Thanks to the nonvolatile nature and logic learning capability, average power dissipation per cycle of RS-based adders goes down with computing cycles. Compared with the state-of-the-art 15-nm CMOS technology, “iMemComp” architecture enables a 60.3% reduction in power dissipation after 10^5^ cycles. Additionally, leakage power dissipation is inherently zero in “iMemComp” using nonvolatile RS devices, whereas CMOS transistors face severe leakage power issues. (**b**) Comparison of computing speed of 32-bit adder circuits based on RS devices and 15-nm CMOS. Parallelism of ‘iMemComp’ is defined as the number of bits involved in the parallel adding based on crossbar arrays. Large-scale circuit simulation shows that the equivalent computing speed improves significantly with higher degree of parallelism. Compared with high-performance 15-nm CMOS circuits, a 76.8% improvement can be achieved. Theoretically, aggressive speed boosting up to femtosecond level may be possible with massively parallel implementation in large-scale arrays. (**c**) Area comparison of RS-based and CMOS-based circuit units. With the complexity of a CMOS circuit increases, the circuit area grows dramatically. However, the increase in the area of RS-based circuits is moderate, and the circuit topology does not change with different functions. The area of a RS-based 32-bit adder is less than 1/700 of a CMOS adder’s area.

From a perspective of power dissipation, today’s CPU performance is around the order of 100 Giga-operands/sec, and a 30 times increase over the next 10 years would boost this performance to 3 Tera-operands/sec, which finally requires 576 Tera-bits (assuming 64-bit per operand) to be moved each second from memories to processors^4^. Considering the energy consumed due to data movement in an order of 0.1 pJ/bit, moving 1 mm on average (10% die size) consumes almost 58 W. For “iMemComp” where no such communication between memory and CPU exists, this is the first big part of energy that can be saved. Owing to the unique features introduced by RS devices into this architecture, large-scale computing tasks are no longer conducted by CMOS processors with a large amount of energy-consuming repetition. Instead, RS arrays can learn user-defined logic functions for the constant reuse, which is energy-efficient for massive data processing since read power is much lower than write power for RS devices^9^,^10^,^11^,^12^. Figure 5a shows the comparison between RS-based circuits and the state-of-the-art 15-nm CMOS technology predicted by International Technology Roadmap for Semiconductors^26^. The comparison focuses on the core modules for computation. Thus, peripheral control circuitry required by RS arrays and external buffer and register circuits required by CMOS circuits are not included in the comparison. The overall power dissipation of RS-based 32-bit adder circuit is directly obtained from transient circuit simulations taking into account interconnect wire resistance and capacitance (Supplementary Table S1). Then, power per cycle can be calculated with the number of computing cycles. Larger scale of computing tasks leads to lower power per cycle in ‘iMemComp’ (Fig. 5a), since adding function can be learned after the first cycle and the read power begins to dominate the average power per cycle with more computing cycles involved. RS-based 32-bit adders can achieve 60.3% reduction in average power dissipation per cycle (Fig. 5a) compared with CMOS circuits after 10^5^ cycles, which is a small amount of computation^4^. Additionally, zero stand-by power in nonvolatile RS arrays would enable normally-off systems and benefit the battery life.

From a perspective of computing speed, parallel structure is able to boost the data processing in ‘iMemComp’. Figure 5b shows the speed comparison between RS-based adders and CMOS-based adders predicted by ITRS^26^. The impact of parallelism, which is defined as the number of bits involved in the parallel adding, is evaluated based on the RS array simulations. Higher degree of parallelism leads to larger number of array rows involved in the computation, with a fixed pulse-train duration to finish the operation. Therefore, the equivalent computing speed improves significantly with higher degree of parallelism (Fig. 5b). Compared with 15-nm high-performance CMOS circuits, a 76.8% improvement in the speed can be achieved by RS-based circuits. Furthermore, theoretical analysis indicates the possibility of femtosecond-level computing speed under a higher degree of parallelism (Fig. 5b), which may require robust design of large-scale crossbar RS arrays.

From a perspective of circuit area, the highly compact crossbar structure of “iMemComp” systems eliminates the complex routing and layout that are necessary for CMOS-based logic circuits, and is able to achieve the smallest possible cell area (4 *F*^2^/cell), where *F* is the minimum feature size allowed by lithography. Figure 5c shows the area comparison between RS-based circuits and CMOS circuits^26^. For more complex circuits, the area increase for RS circuits is more moderate (Fig. 5c) than that of CMOS circuits, since the reconfigurable logic in ‘iMemComp’ does not need complex design of circuit topology, layout and routing. Compared with the CMOS solution, the area of RS-based 32-bit adders can be reduced by over 700 times.

As a non-von Neumann architecture, “iMemComp” capitalizes on both device-level and circuit-level properties for parallel computing, and the reconfigurable logic learning architecture mimics the way human brain works in terms of learning knowledge through practice and accepting new ideas. The experimentally demonstrated nonvolatile logic and memory features together with superior performance in power, speed and area have proven the feasibility of high-density, massively parallel, ultra-low-power information processing systems with memory and logic unified by single-type devices. The important lesson we have learned from this research, is that one should explore the use of novel device properties in architectural innovations and fully exploit the computational potential of emerging technologies for the increasing demand of our information society.

## Methods

### Device fabrication and packaging

Fabrication of Pt/HfO_x_/Ti/TiN resistive switching devices was performed without high thermal budget process. Firstly, a 20-nm Ti adhesion layer and a 50-nm Pt bottom electrode were prepared on an 8-inch silicon substrate by electron beam evaporation. Then, 4-nm HfO_x_ was deposited by reactive sputtering in argon and oxygen ambient. After that, thin Ti capping layer of 2 nm was sputtered. After a 50-nm TiN top electrode was deposited by reactive sputtering in high vacuum and patterned with 248 nm lithography, dry etch was performed to form the square-shape devices. Finally, post-metal dielectrics of plasma enhanced chemical vapor deposition (PECVD) SiN/SiO_2_ and Al metallization were used to complete device fabrication. The fabricated devices were packaged using dual inline-pin package (DIP) technique.

### Electrical measurements

The device electrical measurements were performed using an Agilent B1500A semiconductor parameter analyzer together with a Cascade Probe Station. During device measurements, the top electrode TiN was applied with voltage source and the bottom electrode Pt was grounded. The measurements of AOI logic operations were carried out on the packaged test chip using Agilent 93000 SoC Series platform. During testing, pulses for computing were applied at top electrodes (bit lines) and the common bottom electrodes (word lines) in series with a load resistor was grounded.

## Additional Information

**How to cite this article**: Li, H. *et al.* A learnable parallel processing architecture towards unity of memory and computing. *Sci. Rep.* **5**, 13330; doi: 10.1038/srep13330 (2015).

## Supplementary Material

Supplementary Information
