# Supplementary material for: A learnable parallel processing architecture towards unity of memory and computing
Source: Sci Rep. 2015 Aug 14;5:13330. doi: 10.1038/srep13330 (PMC4536493; doi:10.1038/srep13330)
Supplement: Supplementary Information [file srep13330-s1.pdf]

Supplementary Information

A learnable parallel processing architecture towards unity of memory and computing

H. Li[1*], B. Gao[1*], Z. Chen[1], Y. Zhao[1], P. Huang[1], H. Ye[1], L. Liu[1], X. Liu[1] & J. Kang[1†]

Affiliations:

[1]Institute of Microelectronics, Peking University, Beijing 100871, China

[*]These authors contribute equally to this work.

[†]Correspondence to: kangjf@pku.edu.cn

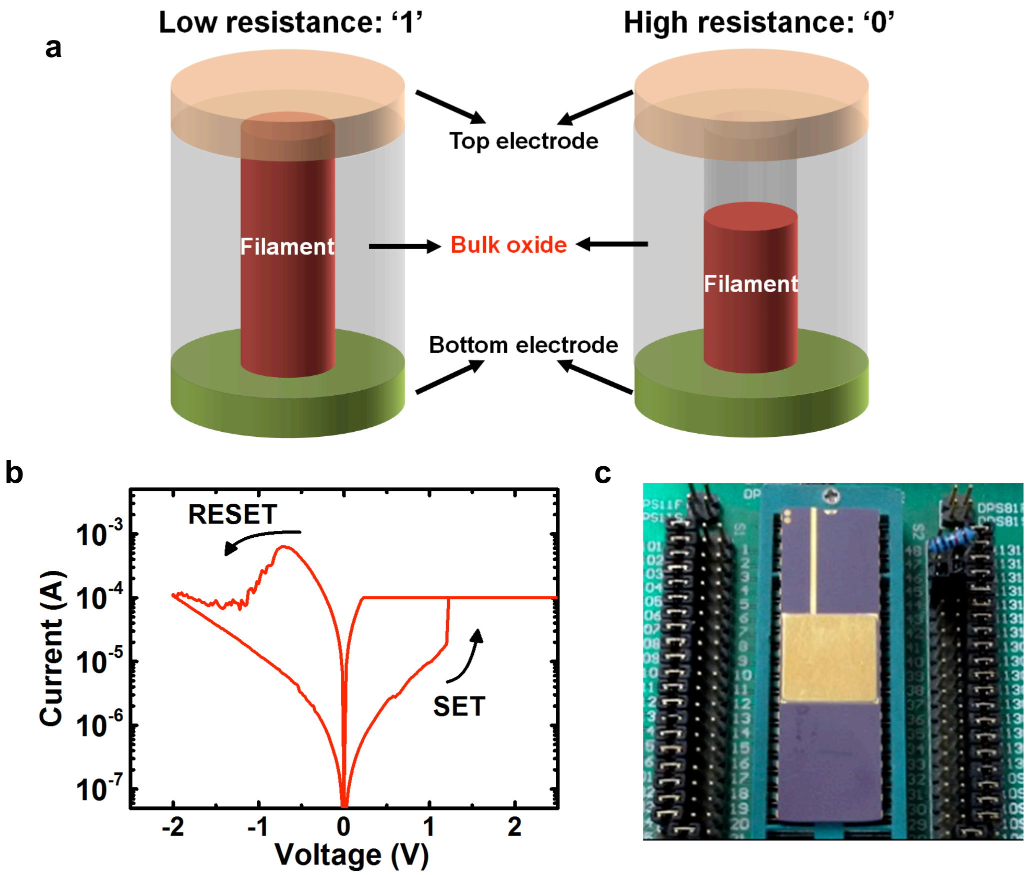

Supplementary Figure S1 | Resistive switching devices. (**a**) Schematic of resistive switching (RS) device structure. In this work, the top and bottom electrodes of RS devices are TiN and Pt respectively. HfO_x resistive switching layer with a thin Ti capping layer is sandwiched between the two electrodes. The formation and rupture of conductive filaments between the electrodes result in the low and high resistance, reespectively. (**b**) Typical measured I-V characteristics of RS devices. By applying a positive voltage, RS devices can be switched from high to low resistance, which is called “SET”. In contrast, by applying a reverse voltage, RS devices can be switched from low to high resistance, which is called “RESET”. The hysteresis curves indicate the memory function of RS devices. (**c**) Photograph of the packaged test chip of RS devices used for the electrical measurements.

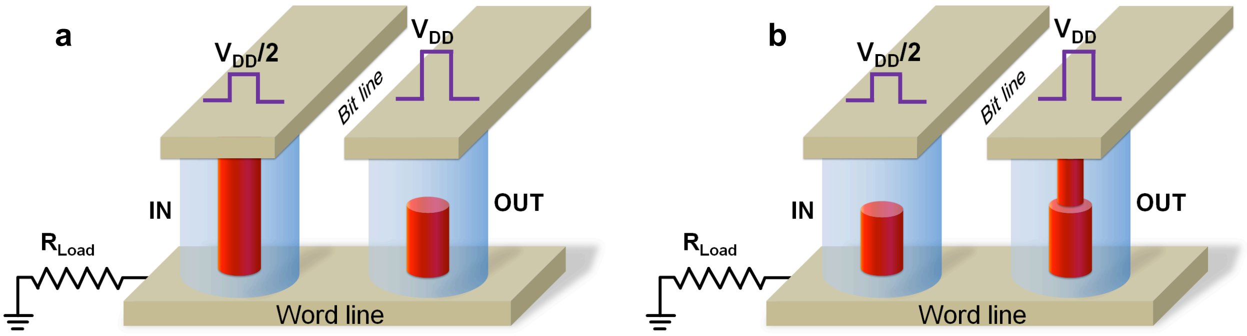

Supplementary Figure S2 | Principle of computing with RS devices. The building block for RS computing is a pair RS devices with pulses ($V_{DD}/2$ and V_{DD}) applied at bit lines. Initially, cells for output (OUT) are in high resistance state (HRS). (**a**) If the cell for input (IN) is in low resistance state (LRS), the voltage dividing between IN and the resistor R_{Load} will raise the electrical potential of the common bit line, which will reduce the voltage drop across the OUT cell, resulting in the voltage lower than the SET voltage threshold. Therefore the OUT cell remains HRS. (**b**) If the IN cell is in HRS, leaving no direct current path from the word line to ground, then the electrical potential of the bit line will remain close to ground. Therefore, the voltage drop across OUT cell is approximate to V_{DD}, which is larger than SET threshold, leading to SET operation and thus, the transition to LRS. The basic logic operation above can be treated as the "state interaction" between RS cells under external pulse train operation.

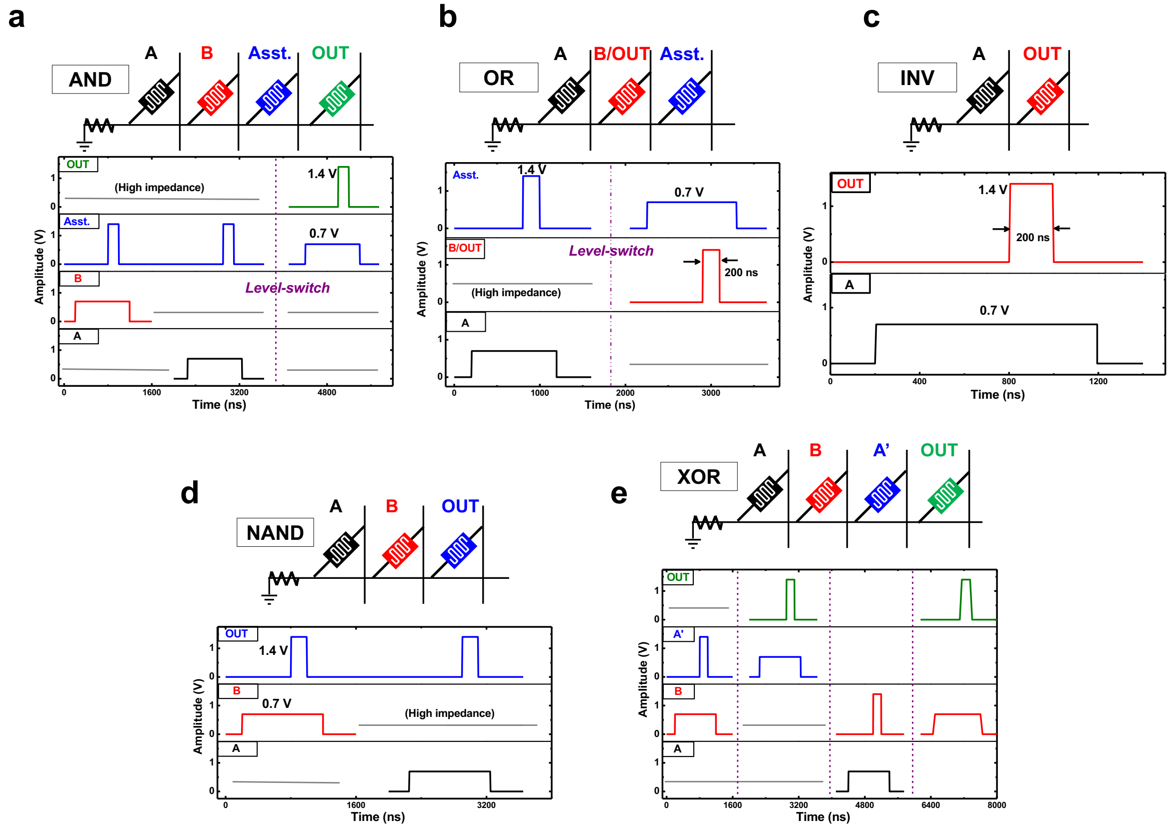

Supplementary Figure S3 | Array configuration and measurement setup of logic operations. (**a**) Array configuration (up) and applied pulse-train waveforms (down) for AND logic operation. Four RS cells ($16F^2$) including one assisting cell are used for computing in total. (**b**) Array configuration (up) and applied pulse-train waveforms (down) for OR logic operation. Three RS cells are used ($12F^2$), where one input cell also serves as output. (**c**) Array configuration (up) and applied pulse-train waveforms (down) for INVERT (or "NOT") logic operation. Two RS cells are used ($8F^2$). (d) Array configuration (up) and applied pulse-train waveforms (down) for NAND operation. Three RS cells are used ($12F^2$). (e) Array configuration (up) and applied pulse-train waveforms (down) for XOR operation. Four RS cells are used ($16F^2$). Under all circumstances, V_{DD} is set to be 1.4 V and half V_{DD} is 0.7 V. Computing time for each cycle is 200 ns due to the interconnect limitation of the measurement platform. For the circuit operations, the speed is determined by the device switching speed as well as interconnect parasitic effects.

Supplementary Note: Physics-Based SPICE Model of RS Devices

It is widely accepted that the resistive switching phenomenon in metal-oxide materials is a result of formation and rupture of conductive filament (CF), which is formed by a chain of oxygen vacancies (V_O). Therefore, the switching characteristics of RS devices are strongly correlated to the CF evolution processes. As illustrated in Supplementary Fig. S4(a), during SET process, oxygen ions (O^{2-}) are generated and V_O are left behind in the oxide layer. This process causes the formation of a CF connecting two electrodes, resulting a transition from HRS to LRS. During the RESET process, under electrical field and Joule heating effect, the O^{2-} reserved in the active electrode will drift back to the switching layer and recombine with the positive charged V_O, which then leads to CF rupture process. Based on this physical picture, a device compact model can be built, as shown in Supplementary Fig. S4(b). The key control variables are the tunneling gap distance (*g*) and the CF radius (*r*), which describe a 2-D filament in the switching layer. During the SET process, the growth rates of CF in length and in radius are described by the two-step process:

$$dg / dt = af \exp\left(-(E_a - \alpha_a ZeE) / k_B T\right)$$

$$dr / dt = \left(\Delta r + \Delta r^2 / 2r\right) f \exp\left(-(E_a - \alpha_a ZeE) / k_B T\right)$$

During the RESET process, the rupture rate of CF is determined by the slower one of two physical processes, namely, O^{2-} release from electrode and V_O/O^{2-} recombination:

$$dg / dt = af \exp\left(-\left(E_i - \gamma ZeV\right) / k_B T\right)$$

$$dg / dt = af \exp\left(-E_h / k_B T\right) \sinh\left(\alpha_h ZeE / k_B T\right).$$

During the switching, the local CF temperature plays an important role to accelerate the temperature-dependent processes incorporated in the equations above. Joule heating affects the local CF temperature and can be described by:

$$T = T_0 + IVR_{th}.$$

The compact model is implemented in HSPICE using Verilog-A. As shown in Supplementary Fig. S4(c), the conduction of RRAM cell is modeled based on two dominant mechanisms: hopping current paths and metallic conduction paths. I-V characteristics associated with *g* and *r* can be calculated as:

$$I_{hop} = I_0 \left(\pi r^2 / 4\right) \exp\left(-g / g_T\right) \sinh\left(V_{gap} / V_T\right)$$

$$I_{CF} = \pi r^2 V_{CF} / 4\rho\left(g_0 - g\right).$$

Finally, we take into account the parasitic effects originating from electrode capacitance (C_p), contact resistance (R_C) and leakage paths (R_p) in the oxide layer (Supplementary Fig. S4(d)). Thereby, transient responses of RS devices in a circuit operation environment can be captured by HSPICE simulations. All the key parameters used in the model is listed in Supplementary Table S1. The model can fit the experimental behaviors of RS devices well (Supplementary Fig. S5).

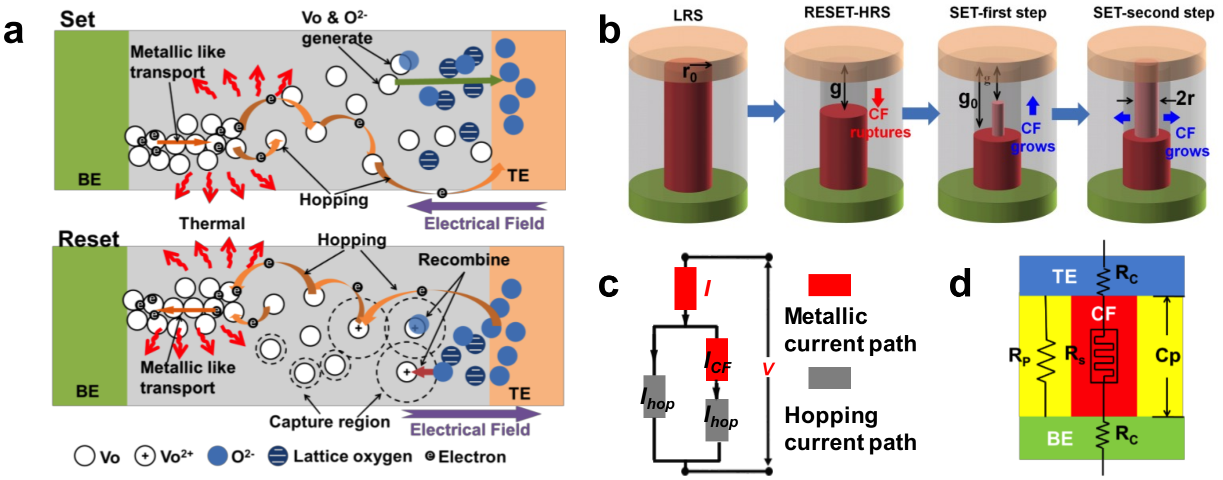

Supplementary Figure S4 | Physics-based SPICE model for circuit simulations. (**a**) Schematic of the switching mechanisms of metal-oxide RS devices. (b) Compact modeling of CF evolutions processes during SET and RESET operations on RRAM cell. Key physical variables are gap distance (g) and CF radius (r). (c) Schematic of conduction paths in RRAM, which helps to define basic elements in the Verilog-A compact model. (d) Parasitic elements of the MIM-structure RRAM. 'R_s' element stands for the core model illustrated in (c).

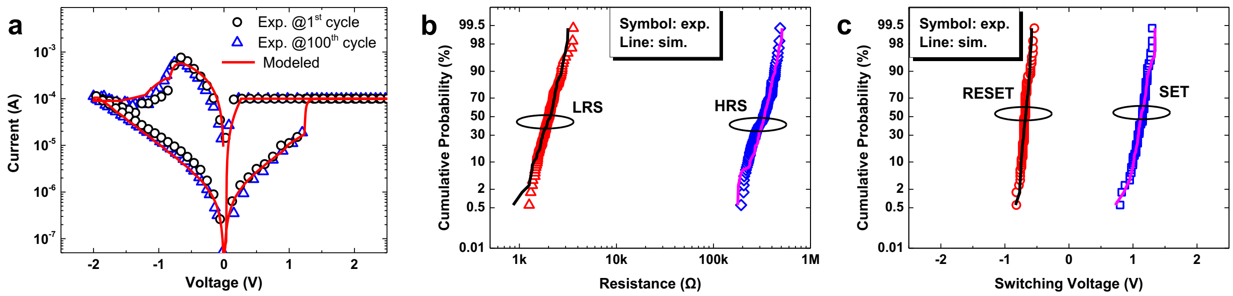

Supplementary Figure S5 | Model verification with electrical measurements. (**a**) Measured (symbol) and simulated (line) I-V characteristics of HfO_x-based RS devices fabricated in this work. The model can well reproduce the I-V behaviors of RS devices. (**b**) Measured (symbol) and simulated (line) statistical distributions of LRS and HRS states. (c) Measured (symbol) and simulated (line) statistical distributions of switching voltages for SET and RESET operations. The model is verified by the key experimental behaviors of RS devices, and is used in HSPICE (a commonly used circuit simulators with golden-standard accuracy) for the circuit simulations of RS-based adder circuits.

Supplementary Table S1

Parameters of Resistive Switching Device Model

Parameters & Values		Descriptions
I_0	10 μA/nm^2	hopping current density in the gap region
ρ	19.64 μΩ·m	resistivity of the CF
a	0.25 nm	distance between adjacent oxygen vacancy (V_O)
f	10^{13} Hz	vibration frequency of oxygen atom in V_O
E_a	0.7 eV	average active energy of V_O
E_h	1.1 eV	hopping barrier of oxygen ion (O^{2-})
E_i	0.8 eV	energy barrier between the electrode and oxide
α_a & α_h	0.75 nm	enhancement factor in lower E_a & E_h
γ	1.5	enhancement factor of external voltage
Z & e	1 & e	charge number & unit charge
Δw	0.5 nm	effective CF extending width
R_{th}	5×10^5 K/W	effective thermal resistance
R_H	200 MΩ	parasitic resistance between electrodes
R_L	20 Ω	parasitic contact resistance of electrodes
C_P	20 fF	parasitic capacitance between electrodes
R_{wire}	12.78 Ω	interconnect wire resistance per cell
C_{wire}	0.046 fF	interconnect wire capacitance per cell

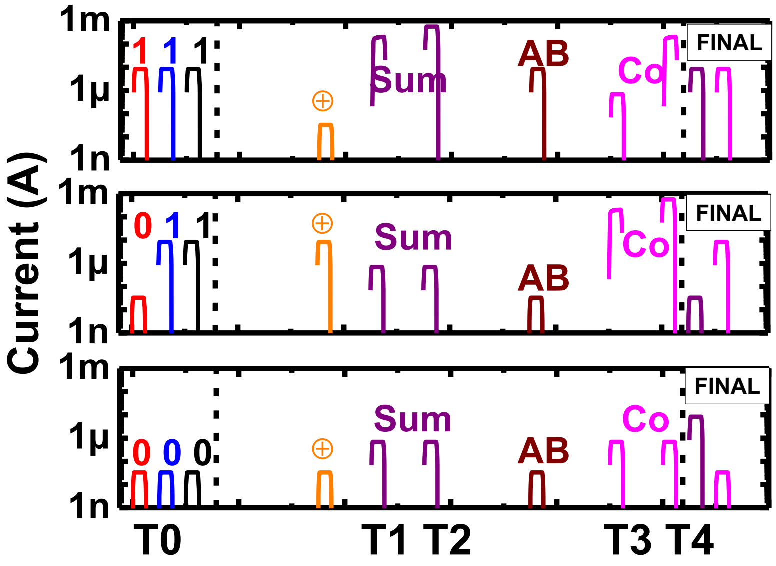

Supplementary Figure S6 | Simulated full adder operation from a perspective of cell current. Corresponding to **Fig. 4a** and **Fig. 4b** in the main text, here show the simulated current values across different cells in the array during the parallel computing process. Along the computation we can get the logic results of AND, XOR, and ADD. The initial and final readout correspond to the gray-scale map in **Fig. 4b**.

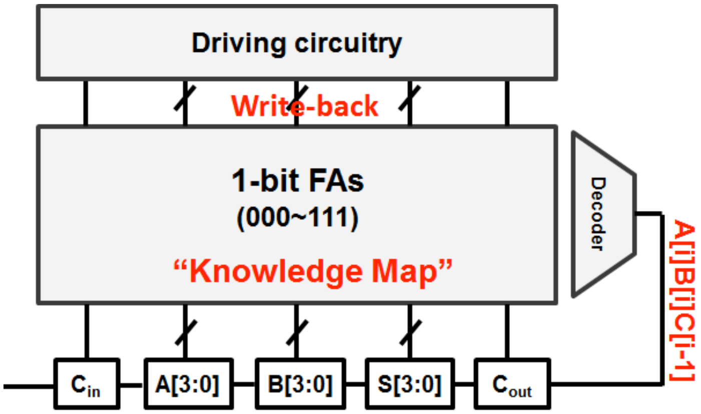

Supplementary Figure S7 | Circuit diagram of a 4-bit adder. The memorized "knowledge map" from logic learning operations can be used for multi-bit computing. For 4-bit adding, each bit *A[i]B[i]C[i-1]* is used as the input for an 3-8 decoder. Since for 1-bit adding operation there are eight input combinations in total, one row out of eight will be selected to obtain the stored data back. After computing, results are stored *in situ* for the 4-bit adder.

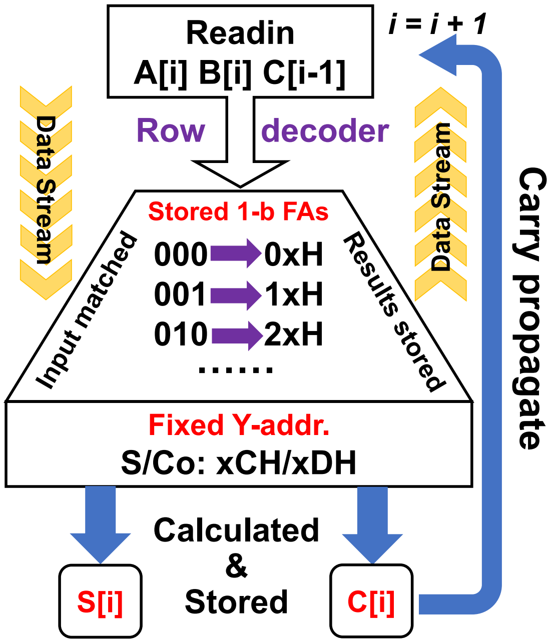

Supplementary Figure S8 | Flow of logic learning for multi-bit computing. Multi-bit logic operations are based on the logic learning results. Taking multi-bit adder operation as an example, we first read in the input combination *A[i]B[i]C[i-1]* as the decoding input. Then, the row decoder selects the row in the array where previously logic learning is finished and matched data are stored. Next, through column selection we can find the stored output *S[i]* and *C[i]*. *S[i]* is written back and *C[i]* is used in the repeated carry propagation until all the bits finish computing.
